# Mechanism of inflammasomes in cancer and targeted therapies

**DOI:** 10.3389/fonc.2023.1133013

**Published:** 2023-03-20

**Authors:** Qingdan Gu, Jiazhen Zou, Ying Zhou, Qiuchan Deng

**Affiliations:** ^1^ Department of Clinical Laboratory, Shenzhen Longhua District Central Hospital, Guangdong Medical University, Shenzhen, Guangdong, China; ^2^ Department of Laboratory Medicine, Shenzhen Second People’s Hospital, The First Affiliated 5 Hospital of Shenzhen University, Health Science Center, Shenzhen, China

**Keywords:** inflammasomes, NOD-like receptors (NLRs), cancer, targeted therapeutics, natural extracts, synthetic small molecule targeted drugs

## Abstract

Inflammasomes, composed of the nucleotide-binding oligomerization domain(NOD)-like receptors (NLRs), are immune-functional protein multimers that are closely linked to the host defense mechanism. When NLRs sense pathogen-associated molecular patterns (PAMPs) and damage-associated molecular patterns (DAMPs), they assemble into inflammasomes. Inflammasomes can activate various inflammatory signaling pathways, including nuclear factor kappa B (NF-κB) and mitogen-activated protein kinase (MAPK) signaling pathways, and produce a large number of proinflammatory cytokines, which are closely associated with multiple cancers. They can also accelerate the occurrence and development of cancer by providing suitable tumor microenvironments, promoting tumor cell proliferation, and inhibiting tumor cell apoptosis. Therefore, the exploitation of novel targeted drugs against various inflammasomes and proinflammatory cytokines is a new idea for the treatment of cancer. In recent years, more than 50 natural extracts and synthetic small molecule targeted drugs have been reported to be in the research stage or have been applied to the clinic. Herein, we will overview the mechanisms of inflammasomes in common cancers and discuss the therapeutic prospects of natural extracts and synthetic targeted agents.

## 1 Introduction

Inflammasomes are a critical component of the innate immune system and play an essential role in defending against invasion by external xenobiotics. Unlike adaptive immunity, innate immunity is a nonspecific immunity in which immune cells have pattern recognition receptors (PRRs) that recognize pathogen-associated molecular patterns (PAMPs) from pathogenic microorganisms and damage-associated molecular patterns(DAMPs) from tissue damage to activate the immune response (1, 2). PRRs are distributed not only on the cell membrane but also in the cytoplasm. The membrane PRRs are composed of Toll-like receptors (TLRs) and C-type lectins (CTLs), while the cytoplasmic PRRs consist of the nucleotide-binding oligomerization domain(NOD)-like receptors (NLRs), retinoic acid inducible gene-I (RIG-I)-like receptors (RLRs) and absent-in-melanoma (AIM)-like receptors(ALRs) (3–5). NLRs and ALRs can form inflammasomes, of which, NLRs dominate. Upon recognition of ligands by NLRs, it combined with the apoptosis-associated speck-like protein (ASC) and pro-caspase-1 to form the inflammasome complex. This inflammasome cleaves inactive pro-caspase-1 into active caspase-1. Caspase-1 further promotes the secretion of the pro-inflammatory cytokines IL-1β and IL-18, which trigger inflammation by recruiting immune cells (e.g., macrophages) and inducing programmed cell death (e.g., pyroptosis) (6).

Inflammation is a defensive process of the body against injury and infection, but sustained activation of the inflammatory response may be associated with tumorigenesis and metastasis (7). The research indicated that long-term chronic inflammation is closely associated with growth inhibition resistance, angiogenesis, immune escape, malignant transformation, and metastatic potential acquisition (8). All of these processes are associated with the formation of inflammasomes. Mutations in genes encoding inflammasomes can affect the expression of nuclear factor kappa B (NF-κB) and mitogen-activated protein kinase (MAPK) signaling pathways, which may ultimately contribute to the formation of cancer (9). The occurrence of tumors is related to the ability of cancer cells to capture inflammatory signaling pathways and promote their proliferation, migration and invasion (10). However, the exact molecular basis between inflammation and cancer remains unclear. Due to the different pathogenic mechanisms of inflammasomes in different cancers, inflammasomes and related signaling pathways have become a current research hotspot for molecularly targeted therapies. This review highlights the research progress of NLR inflammasomes in common cancers and outlines the potential of inflammasomes and key molecules in signaling pathways as therapeutic targets.

## 2 Structure and classification of the NLR family

**Figure 1:**
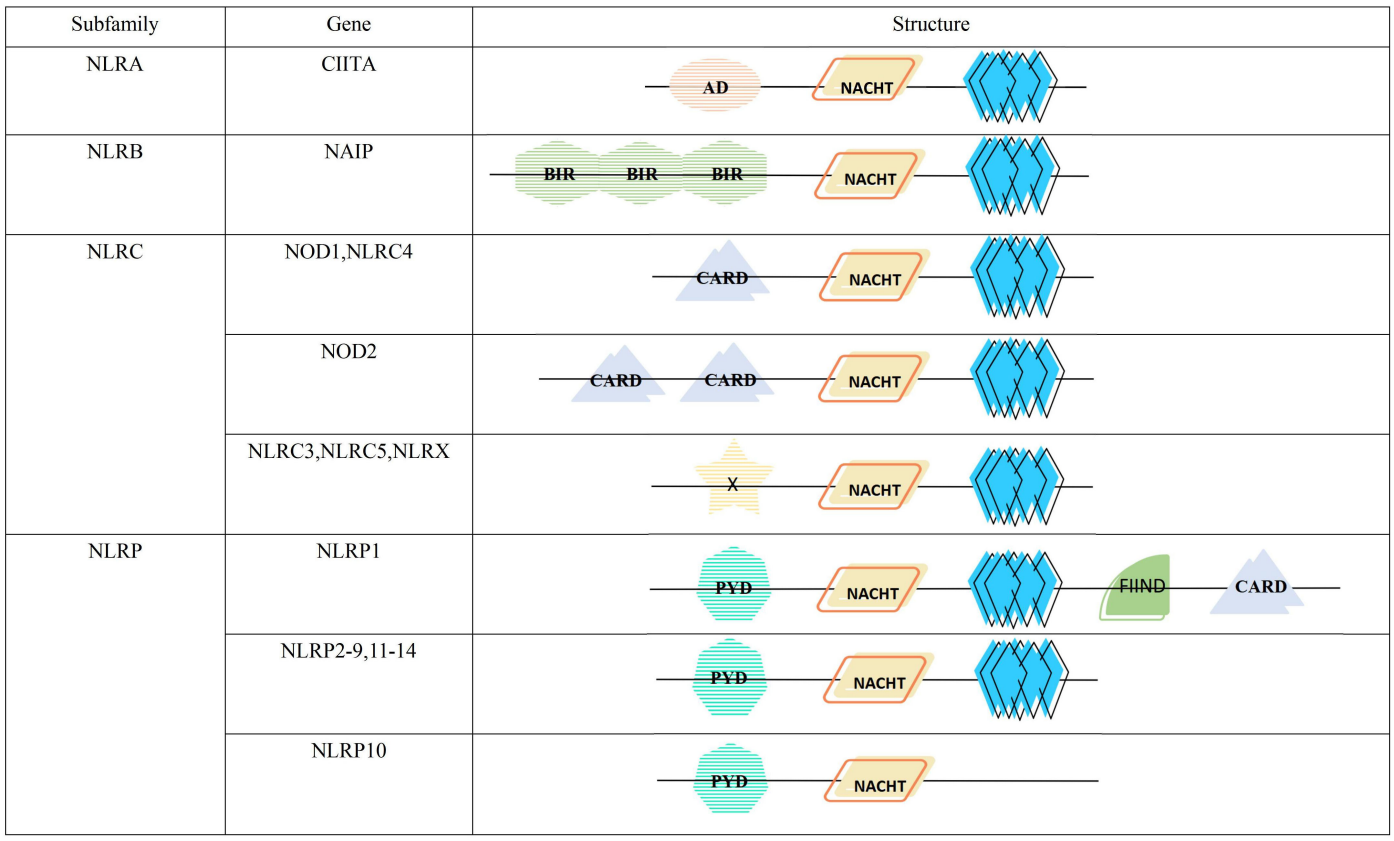
Structure and classification of the NLR family. AD, acidic transactivation domain; NACHT, NACHT domain (consisting of seven distinct con-served motifs, including the ATP/GTPase-specific P-loop, the Mg2+-binding site, and five more-specific motifs); BIR, baculovirus inhibitor of apoptosis repeat; CARD, caspase activation and recruitment domain; X, unidentified; PYD, pyrin domain; FIIND, function to find domain; leucine-rich repeat.

The NLR proteins consist of three distinct parts, the NACHT domain in the central region, flanked by leucine-rich repeats (LRRs) at the C-terminus, and an effector domain at the N-terminus(**Figure 1**). The NACHT domain has dNTPase activity and is involved in ATPase-dependent oligomerization (11, 12). LRRs can sense a variety of afferent signals. For example, NOD1 and NOD2 can detect bacterial peptidoglycan (13), NLRC4 and NAIP sense type III secretion system(T3SS) and flagellin (14), NLRP3 can be activated by sensing other molecules, such as ATP, reactive oxygen species (ROS), silica, uric acid crystals and nigericin (15), but the exact mechanisms by which it senses ligands are not yet clarified. The N-terminal effector domain is used to mediate the signal transduction of downstream targets, thereby activating caspases and various signaling pathways (16).

According to the different N-terminal effector domains, NLRs family is divided into 4 subfamilies, containing a total of 22 members (**Figure 1**). Among them, NLRA subfamily has only one member, the class II major histocompatibility complex transactivator (CIITA), containing the acidic transactivation domain (AD). It can enhance the transcription of MHC class II molecules and inhibits the classical NF-κB pathway (17). NLRB subfamily, characterized by a baculoviral inhibitory repeat-like domain (BIR), is also comprised of a single member, the NLR family apoptosis inhibitory protein(NAIP). It is responsible for preventing apoptosis mainly by inhibiting the activities of caspase-3, caspase-7 and caspase-9 (18). NOD1, NOD2 and NLRC4 with the caspase activation and recruitment domain (CARD), and NLRC3, NLRC5 and NLRX1 with unknown domains all belong to NLRC subfamily. The former can monitor microbial invasion and affect cytokines secretion by upregulating or downregulating inflammatory signaling, while the latter regulates autophagy and cell death by fine-tuning the cascade response of inflammatory signaling and the type I IFN signaling pathway (19). NLRP containing pyrin domain (PYD) has 14 members, namely NLRP1-14, which are mainly responsible for the modulation of inflammatory signaling and apoptosis (20).

## 3 Activation pathways of inflammasomes

Inflammasomes are a set of multi-protein complexes assembled with the participation of natural immune recognition receptors, which function as an inflammatory immune response by secreting inflammatory cytokines and inducing cellular pyroptosis (21). “The canonical inflammasome pathway” was defined when the inflammasome activation was mediated by caspase-1, while “The noncanonical inflammasome pathway” was classified when the inflammasome activation was dependent on human caspase-4/5 or mouse orthologue caspase-11 (22, 23). These two activation pathways and their roles are described in detail below.

### 3.1 The canonical inflammasome pathway

**Figure 2:**
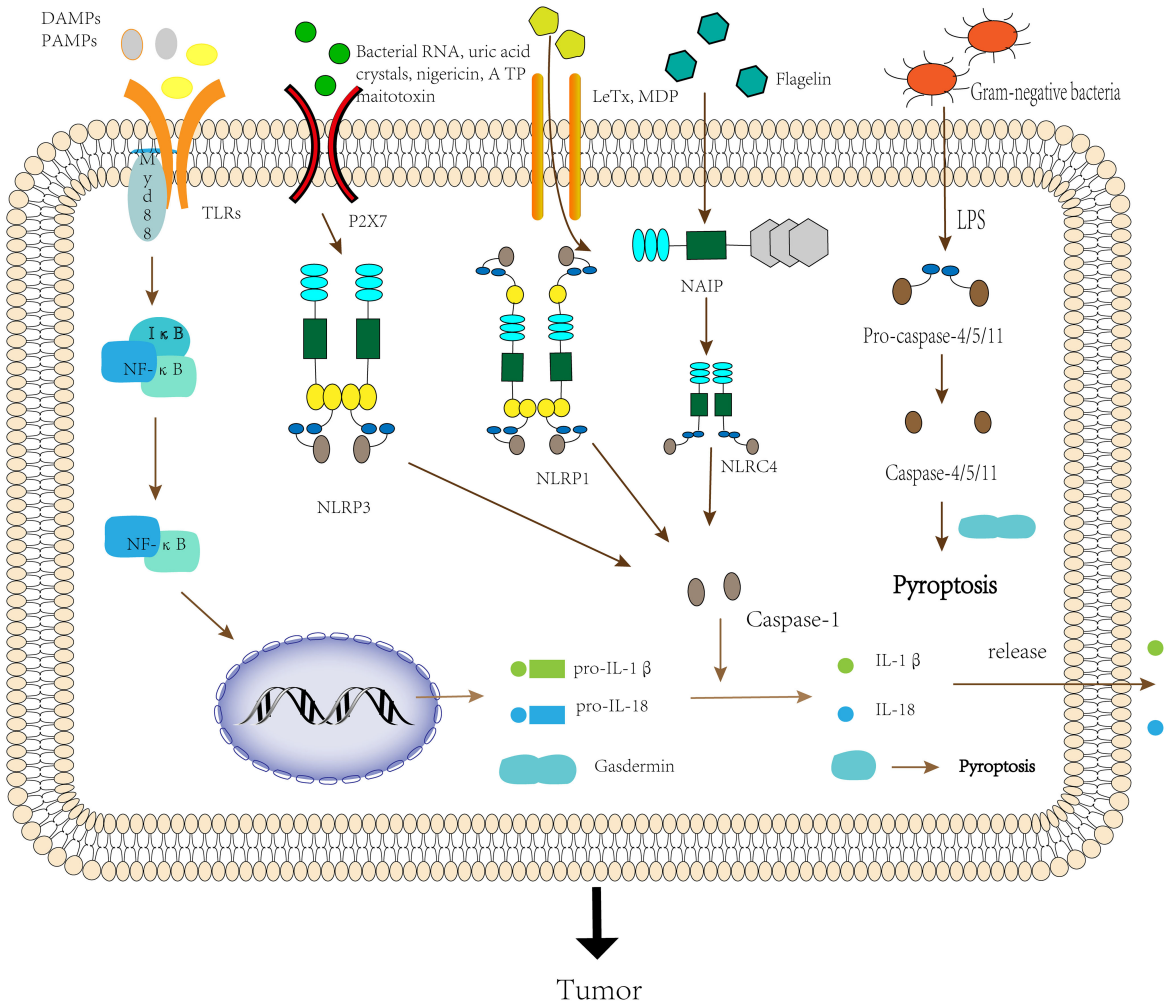
The activation process of the inflammasome.

Activation of the inflammasomes requires the involvement of “activators”. In the absence of activator stimulation such as DAMPs and PAMPs, LRRs interact with the NACHT domain and inhibit inflammasome formation (24). When LRRs detect DAMPs, PAMPs or environmental stimuli in microbes, the environment or the organism, NLR proteins start recruiting the adaptor protein ASC, which interacts through the Pyrin-Pyrin structural domain (25). Then the effector domain pro-caspase-1 binds to ASC through the CARD-CARD domain to assemble into an inflammasome, and finally, oligomerization to form an inflammasome complex possessing a heptamer (26). Since NLRP1 itself possesses a CARD domain, it can form inflammasomes directly through the interaction of the CARD-CARD domain with pro-caspase-1 without the need for ASC (27). Nonetheless, related studies have shown that ASC can enhance the stability of the CARD-CARD domain between NLRP1 and pro-caspase-1, thereby driving the immune response (28). Thus, ASC is an essential component of the inflammasomes complex assembly process, and when the body is deficient in ASC, it can lead to a variety of diseases (29, 30). NLRC4 does not contain a Pyrin structural domain, and it functions primarily by interacting with NAIP to form NAIP-NLRC4 inflammasomes. First, ligands such as flagellin or bacterial type III secretion system (T3SS) interact with non-activated NAIP, causing a conformational change and thus activation. Then ligands with activated NAIP further cause a conformational change in non-activated NLRC4, and activated NLRC4 can recruit more NLRC4 monomers to form NAIP-NLRC4 inflammasomes (31). Inflammasome assortment can further mediate the formation of active caspase-1 by self-hydrolysis of inactive pro-caspase-1, which can cleave pro-IL-1β and pro-IL-18 to form the mature inflammatory cytokines IL-1β and IL-18 (32). Inflammatory cytokines recruit immune cells, such as T lymphocytes and neutrophils, at the sites of infection and inflammation, thereby regulating the innate and adaptive immune response. They also initiate autocrine and paracrine inflammatory signaling cascades, providing survival signals to normal cells to prolong their lifespan and death signals to abnormal cells to accelerate their death (33). In addition, activated caspase-1 cleaves gasdermin D (GSDMD) and releases its N-terminal domain, which translocates to the cell membrane to form a pore that mediates the release of cellular contents such as IL-1β and IL-18, and induces pyroptosis (34) (**Figure 2**).

### 3.2 The noncanonical inflammasome pathway

The noncanonical inflammasome pathway is triggered by caspase-4/5/11. When TLR4 of the organism senses the lipopolysaccharide(LPS) of Gram-negative bacilli, it promotes the transcription of pro-IL-1β and pro-IL-18 by inducing the activation of NF-κB (35). In addition, when signaling to Trif/IRF3 induces the expression of type-I-IFNs, which promotes the secretion of pro-caspase-11. But it remains unknown whether the activation of caspase-11 is due to other additional signals or because pro-caspase-11 is automatically activated after reaching a certain threshold (36). Activated caspase-11 activates the NLRP3 inflammasome to induce caspase-1-dependent maturation and secretion of IL-1β and IL-18, but the activation pathway remains to be determined. In addition, caspase-11 can also cleave GSDMD to induce caspase-1-independent pyroptosis (21). Excessive pyroptosis can lead to a variety of cancer-related diseases, such as gastric cancer, colorectal cancer, and ovarian cancer (37) (**Figure 2**).

## 4 Role of NLR inflammasomes in tumor development and metastasis

Many studies have demonstrated that abnormal activation of inflammasomes is closely associated with several human diseases, for instance, gout, silicosis, periodic fever syndrome, Crohn’s disease, type 1 diabetes, and so on (6, 38, 39). Currently, the increasing evidence shows that the inflammatory response mediated by NLR inflammasomes is involved in regulating physiological processes such as tumor development and metastasis. When the inflammasomes are abnormally activated, a variety of inflammatory cytokines and chemokines, including TNF-α, IL-1β, IL-6, IL-18, CCL2/MCP-1, are excessively secreted, which affect the development of cancer (40, 41). Among them, TNF-α plays a pro-tumor role by promoting DNA damage and inhibiting DNA repair (42). The expression of IL-1β and IL-18 is significantly elevated in a variety of malignancies, and these cytokines can promote cancer development and distant metastasis by triggering the secretion of VEGF, FGF2 and STAT3 (43, 44). IL-6 can promote the invasive ability of cancer cells, which leads to tumor metastasis. But there are some studies with contradictory findings, probably because inflammasomes not only promote tumor progression, but also correlate with apoptosis of tumor cells (45). The following will review the effects and mechanisms of inflammatory response mediated by NLR inflammasomes on common tumors including colorectal cancer, breast cancer, liver cancer and melanoma.

### 4.1 NLR inflammasomes and colorectal cancer

Colorectal cancer (CRC) is a malignant tumor occurring in intestinal epithelial cells, and the most common pathological carcinoma is adenocarcinoma. The overall incidence rate ranks in the top three among malignant tumors, and the mortality rate ranks the fourth, after lung, liver, and stomach cancers (46, 47). Reducing the prevalence and mortality of CRC has become an urgent issue to be solved. At present, surgical treatment, neoadjuvant chemoradiotherapy and adjuvant chemotherapy are the main clinical treatment methods. With the development of endoscopic technology and various auxiliary technologies, the 5-year survival rate of early-stage CRC can be as high as 92% (48, 49). However, there is still no effective treatment to reduce the prevalence. The search for new treatment methods and new therapeutic targets is currently a hot topic in academic circles.

Colorectal cancer model is the most studied cancer model, and a number of studies have shown that a variety of NLR inflammasomes are closely related to it, among which NLRC3, NLRC4, NOD2, NLRP1, NLRP6, NLRP12 inflammasomes have protective effects on colorectal cancer, while the effects of NLRP3 and NOD1 inflammasomes are still controversial (50). Rajendra’s team (51) found that NLRC3 inflammasomes modulated the multiplication and differentiation of stem cells and could promote apoptosis. What’s more, the upregulation of *Nlrc3* was negatively correlated with the occurrence of tumors. In contrast, in *Nlrc3* downregulated enterocytes, cell proliferation was not controlled due to the inability to inhibit the activation of the P13K-mTOR signaling axis, which may lead to tumorigenesis (52). Similarly, in a mouse model of colorectal cancer, the expression of *Nlrc4* mRNA was diminished (50). Compared with wild-type mice, *Nlrc4^-/-^* mice could promote the proliferation of colonic epithelial cells and inhibit apoptosis of tumor cells, thereby promoting tumor formation, suggesting a protective effect of NLRC4 inflammasomes on colorectal carcinogenesis (53). In addition, Allam’s team (54) concluded that the higher incidence of colorectal cancer in *Naip^-/-^* mice may be related to the inability of *Naip^-/-^* mice to suppress the overactivation of STAT3, a transcription factor that promotes tumor growth. STAT3 hyperactivation was not observed in *Nlrc4^-/-^* mice, demonstrating that the protective effect of NAIP inflammasomes may be independent of NLRC4 inflammasomes. Couturier et al. (55) found an increased distal colon tumor load in *Nod2^-/-^* mice by comparison with wild-type mice. This phenomenon may be due to an imbalance of pro- and anti-inflammatory cytokines and a loss of autophagy and apoptosis, leading to chronic inflammation as well as an increased risk of cancer (56). Nashir (57) and his team concluded that NOD2 inflammasomes could inhibit CRC by blocking IRF4, thereby downregulating NF-κB and MAPK signaling pathways. NLRP1 and NLRP6 inflammasomes prevent CRC by mediating the production of IL-1β and IL-18 through caspase-1 (58–60). In addition, it has been shown that CCL5-induced inflammation can increase the number of tumors in *Nlrp6^-/-^* mice (61). The team of Zaki (62) found that NF-κB, ERK, and STAT3 signaling pathways were highly activated and that tumor incidence was increased in the colon of *Nlrp12^-/-^* mice. The ERK pathway activates the oncogenic transcription factor cMyc, which activates multiple oncogenic factors including COX. STAT3 regulates the pro-inflammatory cytokines IL-17 and IL-23, the anti-apoptotic protein Bcl-xL, and a variety of growth factors. Allen (11) also found that classical and non-classical NF-κB signaling pathways were activated and the body was highly susceptible to colorectal cancer in *Nlrp12^-/-^* mouse models. Among them, NLRP12 interacted with NIK in the non-classical NF-κB pathway and inhibited the production of p52, making this pathway dysregulated. Compared to the non-classical NF-κB pathway, the classical pathway has a relatively weak effect. In addition, NLRP12 inflammasomes also was able to regulate MAPK and AKT pathways, resulting in reduced tumorigenesis in mouse models (62). Stefanie’s group (63) found an interesting phenomenon in that NLRP5 inflammasomes expression was not detected in normal intestinal tissues, but it expressed in tissues with intestinal cancer, which indicated that NLRP5 inflammasomes expression was associated with tumorigenesis. However, the specific mechanism of action between NLRP5 inflammasomes and CRC needs to be further explored. The occurrence of CRC is probably related to immunological factors such as the activation of immune response and recruitment of immune cells. NLRP3 was found to be expressed in both immune cells and epithelial cells of colon cancer, manifesting that NLRP3 inflammasomes are involved in the formation of CRC (64). However, this aspect of research is still controversial: some studies have shown a positive correlation between NLRP3 inflammasomes and CRC, but a part of the research holds the opposite view, they suggest that NLRP3 inflammasomes may inhibit tumor development (65). Zaki et al. (66) found that NLRP3 inflammasomes lacking the adaptor proteins ASC and caspase-1 played a protective role in CRC compared to wild-type mice. Another study demonstrated that the use of NLRP3 inflammasomes small molecule inhibitors reduced the incidence of CRC (67). In addition, it has been reported that NOD1 inflammasomes deficiency caused damage to the intestinal epithelial barrier, increased apoptosis, and release of inflammatory cytokines in mouse models, leading to the development and progression of CRC (68, 69). Conversely, some other authors have argued that NOD1 is highly expressed in CRC mice and patients, and increased NOD1 expression would decrease the long-term survival of patients. NOD1 inflammasomes enhances tumor cell adhesion, migration and metastasis by activating MAPK signaling pathway (70). Tumorigenesis and metastasis require an appropriate microenvironment, and Charles et al. (71) suggested that NOD1 expression on myeloid cells formed a microenvironment conducive to tumor growth and may contribute to the development of CRC (**Table 1**).

**Table 1:** Effects of NLR inflammasomes in cancer.

Cancer	NLR inflammasomes	Effect	References
Colorectal cancer	NLRC3	Protection	(51, 52)
	NLRC4	Protection	(53)
	NOD2	Protection	(56, 57)
	NLRP1	Protection	(60)
	NLRP6	Protection	(58, 61)
	NLRP12	Protection	(62)
	NLRP3	Protection	(65, 66)
	NLRP3	Aggravation	(64)
	NOD1	Protection	(68, 69)
	NOD1	Aggravation	(70, 71)
Breast cancer	NLRC4	Aggravation	(72)
	NAIP	Aggravation	(73)
	NLRP3	Protection	(74)
	NLRP3	Aggravation	(75, 76)
	NLRP1	Aggravation	(77)
	NOD1	Protection	(78, 79)
	NOD2	Protection	(80)
Liver lung	NLRP3	Protection	(81)
	NLRC5	Aggravation	(82)
	NLRP12	Protection	(83, 84)
Melanoma	NLRP1	Aggravation	(85)
	NLRP3	Aggravation	(86)
	NLRC4	Protection	(87)
	NLRC4	No effects	(88)

### 4.2 NLR inflammasomes and breast cancer

Among female malignancies, breast cancer ranks first and has become one of the major public health problems in the world (89, 90). With the increasing improvement of breast cancer treatment methods, in addition to traditional surgery, adjuvant chemoradiotherapy and hormone therapy, targeted gene therapy and immunotherapy have also been used in clinical practice. Although the 5-year survival rate of patients has improved significantly, the mortality rate is still high (91, 92). It is of great clinical importance to find new therapeutic targets and develop new preventive and therapeutic measures to reduce the morbidity and mortality of breast cancer.

There have been many studies showing that abnormal expression of multiple NLR inflammasomes are closely connected with breast cancer. The NLRC4/IL-1β signaling pathway has been shown to promote the progression of breast cancer. In adipocytes, NLRC4 inflammasomes induce tumor infiltration into myeloid cells, and IL-1β promotes vascular endothelial growth factor A (VEGFA) secretion and angiogenesis, thereby driving disease progression (72). A study showed that NAIP was expressed in breast cancer and at significantly higher levels than in tumor control groups. In addition, NAIP was overexpressed in patients with poor outcomes and poor prognoses. These results suggest an aggravating role for NAIP inflammasomes in breast cancer (73). Nour (75) et al. proposed that the NLRP3/IL-1β pathway was strongly connected with the development and metastasis of breast cancer. Compared with normal breast tissue, the expression of *Nlrp3, caspase-1 and IL-1β* genes in the NLRP3 inflammatory pathway was significantly upregulated in mouse and human mammary tumor stroma. Among them, NLRP3 inflammasomes could mediate infiltration of CD11b+Gr1+ immune cells into mammary tumor tissues, and IL-1β could promote mammary tumor progression and tumor cell metastasis to the lung by upregulating adhesion molecules expression in primary tumors and metastatic sites. Guo et al. (76) also made similar findings that IL-1β secretion and tumor cell metastasis to the lung were reduced in *caspase-1^-/-^* and *Nlrp3^-/-^* mice compared to wild-type mice. What’s more, blocking IL-1R with inhibitors inhibited tumor growth and metastasis. The authors posited that this may be because activation of inflammasomes and production of IL-1β could recruit myeloid-derived suppressor cells (MDSC) and tumor-associated macrophages to tumor tissues, creating a favorable microenvironment for tumor metastasis. However, François et al. (74) held the opposite view, arguing that NLRP3 inflammasomes exerted anti-tumor functions by stimulating IL-1β release from dendritic cells. It has been proposed that NLRP1 inflammasomes promotes proliferation, migration and invasion of the breast cancer cell line MCF-7 compared to normal breast tissue. IL-1β, IL-18 and ASC, the pivotal elements of the inflammatory signaling pathway, are upregulated in breast cancer cells (77), but the specific cancer-promoting mechanism of NLRP1 inflammasomes needs to be further verified. Some investigators suggested that NOD1 and NOD2 inflammasomes may also be relevant to the negative regulation of breast cancer. In a study of estrogen receptor(ER)-negative Hs578T cell line, the investigators found that the proliferation rate of cells overexpressing NOD1 was reduced *in vitro*, and a similar phenomenon was seen in NOD2 overexpressed cells, even more significant than the former, which may be the result of activation of MAPK pathway signaling (93). Another *in vitro* cellular experiment showed that in the ER-positive MCF7 breast cancer cell line, NOD1 upregulation promoted RIP2 and caspase-8 mediated apoptosis and decreased estrogen-induced cell proliferation response (94). In addition, NOD1 downregulation enhanced the multiplication of breast cancer cells (80). Therefore, NOD1 inflammasomes was considered to be a tumor suppressor in ER-positive breast cancer cells (**Table 1**).

### 4.3 NLR inflammasomes and liver cancer

Liver cancer is the fifth most common cancer, with a mortality rate of 9.1% of all cancers. It is estimated that there were about 780,000 new cases of liver cancer and nearly 740,000 deaths in 2012 (78). Hepatocellular carcinoma (HCC) and intrahepatic cholangiocarcinoma (ICC) are the two main histological types of liver cancer, of which 70%-85% are HCC and 10% - 20% are ICC (79, 95). There are many causes of hepatocellular carcinomas, such as immune activation due to chronic inflammation, bacterial translocation of intestinal flora and damage to hepatocytes by endotoxins secreted by them, etc. (96, 97). Most researchers now believe that certain genetic mutations, upregulation or downregulation of gene expression due to chronic inflammation caused by hepatitis viruses, such as hepatitis B virus (HBV) or hepatitis C virus (HCV), may further contribute to the development of cancer, which is considered a potential risk factor for hepatocellular carcinoma (98). Significant genetic variants in NLRs were identified in a genomic sequence study, among which the gene mutations of NLRP3, NLRC5, NLRP12 and activation of their signaling pathways were widely noted (99, 100) (**Table 1**).

Raised levels of NLRP3 inflammasomes were observed in patients with chronic HBV infection, indicating that NLRP3 inflammasomes is an important intracellular receptor for HBV infection and that it may induce inflammatory responses by stimulating the secretion of IL-1β and IL-18 (101). In patients suffering HCV, the NLRP3 inflammatory signaling pathway has also been implicated as a factor stimulating elevated serum IL-1β levels (100). In a clinical study, NLRP3 expression was found to be upregulated in hepatitis and cirrhosis but downregulated in HCC (102). Another study found that human stanniocalcin-1 could reduce the volume of HCC tumor tissue by upregulating NLRP3 signaling pathway (103). This implies that NLRP3 inflammasomes are negatively associated with HCC. NLRC5 inflammasomes can promote HCC, it has been shown that knocking out *Nlrp5* can suppress tumor growth and metastasis by targeting the Wnt/β-catenin signaling pathway (104). In addition, NLRP12 inflammasomes also influence the progression of HCC. A proto-oncogene cJun exists in hepatocytes, and JNK, a cJun N-terminal kinase, is prominently activated in hepatocellular carcinoma. NLRP12 inflammasomes can inhibit HCC by suppressing the JNK signaling pathway. Additionally, they can mediate NF-κB downregulation and ERK activation, which has negative regulatory effects on HCC (81). Hepatocyte-specific cytokines include chemokines such as CXCL1, CXCL2 and CCL2, which enhance inflammatory cell infiltration, thereby increasing inflammation in the tumor environment. It has been found that NLRP12 inflammasomes can induce the downregulation of hepatocyte-specific cytokines and exert an inhibitory effect on HCC (82) (**Table 1**).

### 4.4 NLR inflammasomes and melanoma

Melanoma is one of the fastest growing cancers in the world and the most aggressive of all skin cancers, with an increasing incidence worldwide in recent years (83, 84). It is a malignant tumor derived from the malignant transformation of melanocytes and usually occurs in the skin and mucous membranes. Surgery is the radical treatment for most early melanoma, and the treatment of metastatic melanoma includes molecularly targeted therapy and immunosuppressive therapy. However, when it comes to patients who do not respond to immunotherapy, who do not have appropriately targeted drugs, who have relapsed, or who have failed to exhaust their available treatment options, their mortality rate is very high (105). Therefore, finding new therapeutic targets is of great importance to prolong the long-term survival of patients.

NLRP3 and NLRP1 inflammasomes polymorphisms were associated with susceptibility to melanoma in a Swedish case-control study (106). Zhai (107) mentioned in his paper that NLRP1 expression was upregulated in melanoma cells. Moreover, in *Nlrp1^-/-^* metastatic melanoma cells, decreased inflammatory cytokines secretion and NF-κB activity were observed, while increased caspase-2/-9 activity and promoted apoptosis. These phenomena indicated that NLRP1 inflammasomes promote melanoma growth. Similarly, NLRP3 inflammasomes promote melanoma growth by activating caspase-1 and producing IL-1β, which leads to suppression of anti-tumor immunity generated by NK cells and T cells (9, 108). The role of NLRC4 inflammasomes in melanoma is controversial. A study showed that subcutaneous injection of B16F10 melanoma in *Nlrc4^-/-^* mice accelerated tumor growth (85). The authors also observed that the production of inflammatory factors, chemokines and IFN-γ was reduced in the *Nlrc4^-/-^* model, which could be responsible for the accelerated growth of the tumors. Interestingly, another study used the same mouse model, but there was no discrepancy in neoplasm incidence between wild-type and *Nlrc4^-/-^* mice from the same litter (86). This may be due to the fact that littermate mice largely eliminate the effects of confounding factors such as gut microbiota and genetic differences (53) (**Table 1**).

## 5 Targeted therapies targeting the NLR inflammasomes

The excessive activation of the inflammasome has been shown to be directly related to a variety of autoimmune diseases and cancers. Therefore, the development of targeted therapeutic drugs targeting the inflammasomes and key molecules in their signaling pathways is very promising. More than 50 therapeutic agents targeting inflammasomes and key molecules in their signaling pathways are reported to be still in development or already on the market (87). Most of them are inhibitors of NLRP3 and IL-1. Currently, there are two main classes of drugs targeting inflammasomes, namely natural extracts and synthetic small molecule agents.

### 5.1 Natural extracts

Sulforaphane, a compound extracted from cruciferous vegetables, exerts biological effects by inhibiting the activation of NLRC4 and NLRP3 inflammasomes and the secretion of IL-1β (88). Due to its anti-inflammatory and anti-tumor properties, it is currently in clinical trials for the treatment of prostate cancer (109) and breast cancer (88). It has been shown that in 20 patients of recurrent prostate cancer treated with sulforaphane, the time to Prostate-specific antigen (PSA) doubling was longer than that before treatment (6.1 months before treatment vs. 9.6 months during treatment (p = 0.044) (109), indicating that the progression of the disease was slowed down. Andrographolide is a natural diterpene compound isolated from Andrographis paniculate. It inhibits the activation of inflammasomes mediated by NF-κB, TNF-α and mitochondrial autophagy, resulting in reduced secretion of IL-1β and IL-6, thereby delaying the progression of colon cancer and reducing the tumor load (67, 110). GL-V9 is a derivative of baicalein, which degrades NLRP3 inflammasomes by inducing autophagy, and can also delay colon cancer progression and tumorigenesis (111). Fumigaclavine C is a natural toxin derived from the marine-derived Aspergillus fumigatus. Guo (112) et al. suggested that it could reduce the occurrence of colitis and delay the progression of colon cancer in mice by inhibiting the NLRP3 signaling pathway and downregulating TNF-α, IL-1β and IL-17A. Li et al. (113) mentioned that Fumigaclavine C could significantly downregulate the expression of NF-κB, inhibit the MAPK signaling pathway and induce caspases-3, -8 and -9-mediated apoptosis to inhibit breast cancer development, thus it may be used as a targeted therapy for breast cancer. In addition, Caffeic acid phenethylester, a natural component isolated from propolis, has been proposed as a potential therapeutic component. It further inhibits caspase-1 activation and IL-1β production by inhibiting NLRP3, thereby suppressing the development of inflammation (114). Besides the above drugs, many botanical drugs also target NLRP3 inflammasomes, such as Withaferin A (115) and Mangiferin (116). Beyond that, there are a lot of plant extracts that target other NLRs inflammasomes, such as apigenin, a flavonoid, that protects against colitis in mice by inhibiting the NLRP6 signaling pathway, which is currently in clinical trials for CRC and breast cancer (117) (**Table 2**).

**Table 2:** The agents targeting inflammasomes for cancer treatment.

Compounds 1. Natural extracts	Mechanisms of action	Studies in cancer	Status
Sulforaphane	Inhibit the activation of NLRC4 and NLRP3 inflammasomes and the secretion of IL-1β.	Reduce the PSA level of patients with recurrent prostate cancer. Inhibit the proliferation of breast cancer cells in breast cancer patients.	Phase I/II
Andrographolide	Inhibit the activation of NLRP3 inflammasomes mediated by NF-κB, TNF-α and mitochondrial autophagy; Reduce secretion of IL-1β and IL-6.	Reduce the risk of CAC.	Phase I/II
GL-V9	Degrade NLRP3 inflammasomes by inducing autophagy.	Downregulate the CAC tumor number, size and average tumor burden in C57 BL/6 mice model.	Pre-clinical
Fumigaclavine C	Inhibit the NLRP3 signaling pathway and downregulate TNF-α, IL-1β and IL-17A; Downregulate the expression of NF-κB, inhibit the MAPK signaling pathway and induce caspases-3, -8 and -9-mediated apoptosis.	Delay the progression of colon cancer in mice model. Inhibit breast cancer development in mice model.	Pre-clinical
Caffeic acid phenethylester	Inhibit caspase-1 activation and IL-1β production through suppressing NLRP3.	–	–
Apigenin	Inhibit the NLRP6 signaling pathway.	Clinical trials in CAC and Breast cancer patients are Ongoing.	Pre-clinical
2. Synthetic small molecule agents			
MCC950	Inhibit classical and non-classical NLRP3 inflammasome activation.	Because of it’s serious liver toxicity in phase II clinical trials, so it is not currently used in clinical practice.	–
OLT1177	Block the process of caspase-1 activation by inhibiting the NLRP3 inflammasome, ultimately leading to reduced production of the inflammatory factors IL-1β, IL-6.	Reduce infiltration of myeloid-derived suppressor cells and increased CD8+ T cells and NK cells, increase efficacy of metastatic breast cancers.	Pre-clinical
Oridonin	Suppress the interaction between NLRP3 and NEK7, thereby inhibiting NLRP3 activation and oligomerization.	Limit the proliferation of cancer cells in breast and ovarian cancers.	Pre-clinical
Canakinumab	Anti-IL-1β antibody.	Lung cancer mortality is significantly lower in the canakinumab 300 mg group than in the placebo group. And the incident lung cancer is significantly less frequent in the 150 mg and 300 mg groups.	Launched
Anakinra	Block the binding of IL-1R and IL-1.	Decrease IL-17A levels, increase IFN-γ release, increase CD8 infiltration and showed durable tumor stabilization efficacy in mCRC patients.	Launched
Rilonacept	Block the binding of IL-1β and IL-1α.	It is currently approved for the treatment of inflammatory diseases such as CAPS, but not yet for cancer.	Launched
Bevacizumab	Antagonize the P2X7 receptor.	It can significantly prolong the survival time of mCRC patients. Reduce the risk of death by 21% in patients with non-small cell lung cancer.	Launched
AZD9056	Antagonize the P2X7 receptor.	It is currently approved for the treatment of inflammatory diseases such as rheumatoid arthritis and osteoarthritis, but not yet for cancer.	Phase II
Glyburide	Inhibit the activation of NLRP3 inflammasomes; Antagonize the P2X7 receptor.	Because of the risk of hypoglycemia, it has not been used in the treatment of cancer and is mainly used in the treatment of type 2 diabetes.	Launched

### 5.2 Synthetic small molecule agents

In recent years, several small molecules targeted agents have been identified that act directly on inflammasomes with the advantages of greater specificity, lower cost and less invasiveness, and have been of increasing interest to researchers. Here, several NLRP3 inflammasomes inhibitors are introduced. MCC950, was found to specifically inhibit classical and non-classical NLRP3 inflammasome activation pathways in macrophages *in vitro (* 118). However, it has been found to have serious liver toxicity in phase II clinical trials, so it is not currently used in clinical practice (119). OLT1177, another NLRP3 inflammasome inhibitor based on MCC950, not only avoids the occurrence of liver injury mentioned above, but also can treat inflammation-related diseases by inhibiting NLRP3 inflammasome activation (120). It has been shown that patients with Cryopyrin-associated periodic syndromes (CAPS) treated with high concentrations of OLT1177 for 8 consecutive days have no liver injury and a significant reduction in blood inflammatory factors (120). OLT1177 not only plays a role in the treatment of inflammation-related diseases, but also has a promising future in the field of tumor. Inhibition of the NLRP3 inflammasomes blocked the process of caspase-1 activation, ultimately leading to reduced production of the inflammatory factors IL-1β, IL-6. This process has been shown to be related to the reduction of tumor cell proliferation (121). Oridonin is a small molecule derived from Rabdosia rubescens, and current studies have revealed that it can suppress the interaction between NLRP3 and NEK7, thereby inhibiting NLRP3 inflammasomes activation and oligomerization, but it does not affect AIM2 and NLRC4 (122). It has been shown that Oridonin can limit the proliferation of cancer cells in breast and ovarian cancers. Besides, Bay 11-7082 and Tranilast are also currently developed specific NLRP3 inflammasomes targeting inhibitors (123, 124) (**Table 2**).

Currently, three drugs, canakinumab, anakinra and rilonacept, are approved by the US Food and Drug Administration as targeted therapy for IL-1 (125). Among them, canakinumab is an IL-1β antibody, which has been reported to significantly reduce lethality in lung cancer patients (126). In addition, clinical efforts are also underway to study the efficacy of canakinumab in other cancers, such as non-small cell lung cancer (NSCLC) and triple-negative breast cancer (TNBC) (127). Anakinra is a recombinant IL-1 receptor antagonist that exerts biological effects by directly blocking the binding of IL-1R and IL-1. At present, it has been used to treat a variety of diseases such as myeloma and mCRC (128, 129). Furthermore, various clinical trials have proven that its use in combination with other chemotherapeutic agents can significantly improve patient prognosis and increase survival rates (130). Moreover, rilonacept is a blocking receptor that binds both IL-1β and IL-1α, which is currently approved for the treatment of CAPS (131). Given the preliminary therapeutic effects of these approved biologics on NLRP3-related inflammatory diseases, clinical studies and drug development of the aforementioned IL-1 blockers are steadily progressing (**Table 2**).

P2X7 receptor plays an important role in the NLRP3/caspase-1 cascade, which can promote tumor proliferation, migration and invasion (132). Bevacizumab, a P2X7 receptor antagonist, has an inhibitory effect on tumor growth (133). As early as 2004, it has been approved by the United States for the treatment of metastatic CRC(mCRC). At present, it has been approved for a variety of solid tumors, such as non-small cell lung cancer, renal cell carcinoma, and glioblastoma. Studies have shown that compared with chemotherapy agents (irinotecan, fluorouracil and leucovorin) alone, Patients with mCRC treated with bevacizumab combined with chemotherapy had significantly longer survival time (6.2 months vs10.6 months; p<0.001) (134). Another study showed that the combination of bevacizumab and carboplatin plus paclitaxel in patients with non-small cell lung cancer reduced the risk of death by 21%, as compared with chemotherapy alone (135). What’s more, AZD9056 and Glyburide are also the P2X7 receptor antagonist. The former is used to treat autoimmune diseases such as rheumatoid arthritis and osteoarthritis, and the latter is used to treat type 2 diabetes (136) (**Table 2**).

## 6 Future and prospects

This review focuses on the biological effect of inflammasomes and the targeted therapeutic agents that aim at inflammatory signaling pathways in various cancers. In recent years, we have gained a deeper insight into the role of NLR inflammasomes, which go far beyond the simple recognition of and fight against pathogens outside the organism, and have demonstrated that NLR inflammasomes-mediated inflammatory signaling pathways are closely associated with all stages of cancer development and metastasis. Sustained activation of inflammasomes can promote the secretion of inflammatory cytokines, leading to increased immune cell infiltration, thereby changing the tumor microenvironment. In addition, inflammasomes can inhibit T-cell and NK cell-mediated antitumor activity, all of which can accelerate cancer progression. It has been found that inflammasomes also have vital roles in inhibiting tumorigenesis development, which provides a theoretical basis for the development of novel anticancer drugs.

Since the intricate relationship between inflammasomes and various types of cancer is difficult to elucidate, the mechanism of inflammasomes in cancer and whether they can be potential therapeutic targets for cancer have attracted extensive attention. Currently, a variety of natural plants targeting key molecules in the NLR inflammasomes signaling pathway have been developed for the treatment of inflammatory diseases (115, 116, 137, 138). Nevertheless, their exact mechanisms in the activation of inflammasomes and their anti-cancer effects still need further investigation. What’s more, a number of synthetic inflammasomes antagonists and monoclonal antibodies are used to modulate inflammasomes activity, thereby inhibiting tumor development and metastasis (139, 140). One of them, targeting effector cytokines, proved to be less effective therapeutically. It has been found that this approach may affect other key functions of inflammatory cytokines, for example, patients on long-term IL-1β inhibitors may be more susceptible to infections (141). In addition, we need to be alert to the fact that inappropriate molecular therapy may not only lead to an increased susceptibility of the body to infectious and autoimmune diseases but also aggravate the disease by diminishing the body’s anti-tumor immune response.

In clinical practice, resistance to immunotherapy is considered to be a great challenge. In-depth exploration of the mechanism of inflammasomes and immunotherapy drugs may help to solve this mystery. Currently, immune checkpoint inhibitors (ICIs) have been identified as effective anti-cancer therapies, such as anti-PD-1, in which the role of inflammasomes cannot be ignored. In the study on diffuse large B-cell lymphoma (DLBCL), Lu et al. found that NLRP3 inflammasome activation and significantly elevated IL-18 levels in DLBCL tissues promoted CD8+ T cell apoptosis by up-regulating programmed death ligand-1 (PD-L1), thereby exerting immunosuppressive effects (142). This process, to some extent, inhibits the tumor suppressive effect of anti-PD-1 drugs. Another showed that inhibition of the NLRP3 inflammasome significantly enhanced the efficacy of anti-PD-1 drugs (143). Huseni et al. found in a clinical study that interleukin-6 (IL-6) production due to inflammasome activation was associated with anti-PD-L1 resistance and that, compared with Atezolizumab(anti-PD-L1) alone, combined blockade of PD-L1 and IL-6 receptor (IL6R) significantly reduced drug resistance (144). In addition, the activation of NLRC4 inflammasome can exert immunosuppressive effects by promoting the expression of PD-L1 (145). These studies demonstrate a correlation between NLR inflammasome and adaptive resistance to anti-PD-1 checkpoint inhibitor immunotherapy in the treatment of cancer. Therefore, targeted antagonists against inflammasomes have the potential to become a new immunotherapy strategy.

Although many achievements have been made in the study of inflammasomes, many challenges remain, for example: (1) The activators of multiple inflammasomes are still unknown, and the signaling mechanisms activated by different inflammasomes need to be further explored (146). (2) Different inflammasomes play different roles in the same tissue, and the same inflammasomes play very distinct roles in different tissues (147), making the exploration of mechanisms extremely complicated. (3) Studies on the relevance of inflammasomes to tumors are limited and mostly focused on mouse models. (4) Some diseases involve the regulation of multiple NLR inflammasomes, and whether the effect of the combination of these inflammasomes-targeted drugs is superior to the use of a single targeted drug, etc. These mysteries need more researchers to collaborate to unravel them one by one. Exploring the specificity of NLR inflammasomes in tissues or cells, thoroughly uncovering their biological roles and their pathogenic mechanisms in various stages of tumors, and finding possible therapeutic targets will contribute to the development of novel anti-cancer drugs.

## Author contributions

QD, QG, JZ, and ZY make substantial contributions to the conception or design of the work. JZ, and ZY selected extracted relevant papers of this manuscript. QG wrote the manuscript. QD had primary responsibility for final content. All authors contributed to the article and approved the submitted version.
